# Editorial Note: Urinary Exosomal microRNA-451-5p Is a Potential Early Biomarker of Diabetic Nephropathy in Rats

**DOI:** 10.1371/journal.pone.0334987

**Published:** 2025-10-22

**Authors:** The PLOS One Editors

Following the publication of the Expression of Concern on this article [1,2], concerns were raised about partial image overlap in the underlying data published in the S2 File of [2]. Specifically, in the underlying image data provided for Fig 4, the second control group week 9 panel partially overlaps with the second DM group week 9 panel. The partial overlap observed in the underlying data provided in support of the published Fig 4 results raises concerns about the overall reliability of these data and the validity of the Fig 4 results.

The *PLOS One* Editors issue this Editorial Note to provide an update to the Expression of Concern [2] previously issued on this article [1] and to inform readers that in light of the concerns raised with the S2 File in [2], the Fig 4 results of [1] should also be interpreted with caution.
